# Correlation of Visuospatial Ability and EEG Slowing in Patients with Parkinson's Disease

**DOI:** 10.1155/2017/3659784

**Published:** 2017-02-28

**Authors:** Dominique Eichelberger, Pasquale Calabrese, Antonia Meyer, Menorca Chaturvedi, Florian Hatz, Peter Fuhr, Ute Gschwandtner

**Affiliations:** ^1^Division of Molecular and Cognitive Neuroscience, Neuropsychology and Behavioural Neurology Unit, University of Basel, Basel, Switzerland; ^2^Department of Neurology, Hospital of the University of Basel, Petersgraben 4, 4031 Basel, Switzerland

## Abstract

*Background.* Visuospatial dysfunction is among the first cognitive symptoms in Parkinson's disease (PD) and is often predictive for PD-dementia. Furthermore, cognitive status in PD-patients correlates with quantitative EEG. This cross-sectional study aimed to investigate the correlation between EEG slowing and visuospatial ability in nondemented PD-patients.* Methods.* Fifty-seven nondemented PD-patients (17 females/40 males) were evaluated with a comprehensive neuropsychological test battery and a high-resolution 256-channel EEG was recorded. A median split was performed for each cognitive test dividing the patients sample into either a normal or lower performance group. The electrodes were split into five areas: frontal, central, temporal, parietal, and occipital. A linear mixed effects model (LME) was used for correlational analyses and to control for confounding factors.* Results.* Subsequently, for the lower performance, LME analysis showed a significant positive correlation between ROCF score and parietal alpha/theta ratio (*b* = .59, *p* = .012) and occipital alpha/theta ratio (*b* = 0.50, *p* = .030). No correlations were found in the group of patients with normal visuospatial abilities.* Conclusion.* We conclude that a reduction of the parietal alpha/theta ratio is related to visuospatial impairments in PD-patients. These findings indicate that visuospatial impairment in PD-patients could be influenced by parietal dysfunction.

## 1. Introduction

Cognitive decline is common in patients with Parkinson's disease (PD) and may range from mild impairment to overt dementia [1]. The cognitive symptoms are highly relevant as they go hand in hand with quality of life, disease prognosis, and caregiver burden [2]. The cognitive impairment generates far-reaching individual and health economic implications. Cognitive impairment in PD was mainly characterised by executive dysfunction, attentional, memory, and visuospatial deficits [3, 4]. Previous studies showed that visuospatial disturbances are among the first symptoms of cognitive decline to appear in PD [5, 6]. These deficits become more pronounced as the disease progresses [7] and they are independent of the severity of motor dysfunction and of the overall intellectual status. Interestingly, PD-patients with visuospatial deficits or memory impairment show a higher conversion rate to Parkinson's disease dementia (PDD) than individuals with executive deficits [8, 9].

The cause of the visuospatial deficits remains unclear [10]. Pereira et al. [11] showed that patients with Parkinson's disease and mild cognitive impairment (PD-MCI) have a greater grey matter atrophy in both occipitotemporal and dorsoparietal cortices compared to healthy controls. Furthermore, previous research found that these patterns correlate with visuoperceptual and visuospatial abilities. These results are in line with the dual-stream hypothesis of visual processing which differentiates between two linked visual projection systems [12]. The first system expands from the area 17 (primary visual cortex) over the dorsal visual route towards the areas of the upper temporal lobe and the parietal lobe (occipitoparietal projection system). These areas participate in the analysis of visuospatial information such as movement, depth, position, orientation, and 3D characteristics of objects. The second projection system, the ventral visual stream, is responsible for pattern recognition (analysis of shapes, colours, objects, and faces). It connects area 17 to the lower temporal lobe.

Biomarker-based detection might lead to a better understanding of the cause of the visuospatial decline in PD-patients. Slowing of oscillatory brain activity (as measured by EEG and MEG) has been proposed as a surrogate marker of cognitive dysfunction [1, 13–15]. Soikkeli et al. [15] and also Olde Dubbelink et al. [16] demonstrated significantly different patterns in EEG frequencies between PD-patients and healthy controls. The authors found a decrease of beta and alpha activity and an analogous increase of theta and delta activity. In PDD-patients, the results are even more marked. A previous study showed that the alpha1/theta ratio is a reliable marker for PD-MCI [17]. Furthermore, Schmidt et al. [18] found that alpha/theta ratio discriminates Alzheimer's disease patients from healthy controls. The study of Kamei et al. [13] verified a positive correlation between deficient executive functions in PD and frontal EEG slowing. This relationship indicates that the deficits in executive tasks in PD could be due to a frontal dysfunction. Based on these findings, it would be interesting to investigate whether visuospatial abilities are related to parietal and occipital EEG activity in PD-patients.

More precisely, it is hypothesized that PD-patients with a visuospatial deficit manifest an EEG slowing which should be particularly pronounced in the parietal and the occipital lobe, compared to frontal, central, and temporal areas. To avoid confounding with overall cognitive performance the EEG slowing is matched with a test of memory span measures (short-term memory). This association in turn, is expected to be stronger in the frontal lobe compared to central, temporal, parietal, and occipital areas.

## 2. Materials and Methods

### 2.1. Subjects and Clinical Assessments

Participants were recruited between 2011 and 2015 from the outpatient clinic for movement disorders of the University Hospital Basel or through announcements in the Journal of the Swiss Parkinson's Disease Association. Altogether 72 patients with PD participated in the study. The data used in this study were baseline data collected from two studies. The first study was a computer-based, multidimensional and disease specific training of cognition in patients with PD that has already been published [20]. The second study is an ongoing group-based stress management training in patients with PD. Clinical assessment was performed with optimally medicated patients by means of the sum score of the motor section of the Unified Parkinson's Disease Rating Scale (UPDRS) subscale III [21]. Depression was assessed by Beck's Depression Inventory (BDI) [22]. The levodopa-equivalent (LED) was estimated according to Tomlinson et al. [23]. Inclusion criteria for the study were idiopathic PD according to UK Parkinson's disease Brain Bank Criteria [24] and signed informed consent was obtained from patients. Patients were excluded if they had other severe brain disorders and insufficient knowledge of the German language or if the EEG and the neuropsychology measurement were set apart more than 60 days. For this study, the data of 57 patients with PD were included. Fifteen patients were excluded due to a Mini-Mental State Examination (MMSE) score of <24 (*n* = 3), because of undergoing a deep brain stimulation (*n* = 6) or due to insufficient EEG quality (*n* = 6, see below).

### 2.2. Neuropsychological Assessments

Patients were assessed with a comprehensive neuropsychological test battery. The following tests of this battery were used for this study; Clock Drawing Test, Rey-Osterrieth Complex Figure Test (ROCF) copy task [25], Block Design Test [26], and verbal Digit Span forward [27].

The* Clock Drawing Test* was scored according to Thalmann et al. [28]. It is a reliable measure of cognitive dysfunction [29, 30]. The Clock Drawing Test correlates with visuospatial tests like the ROCF and the Block Design [31, 32].

The* ROCF* is a common neuropsychological screening method for visuospatial abilities [33, 34]. Particularly, the copy variant of the task measures visuospatial construction while the delayed variant indicates visuospatial memory performance [34]. In the ROCF, the patients had to copy a complex figure. Afterwards, they had to reproduce it as complete as possible after a delay of 30 minutes. The ROCF was evaluated according to Aebi and Mistridis [35] based on Spreen and Strauss [36]. The sum score ranges from 0 to 36 points. The data were transformed into education and age controlled *z*-scores according to Aebi and Mistridis [35].

*Block Design* is a subtest of the revised Hamburg Wechsler Intelligence Scale for Adults [26]. The patients received at the beginning 4 and later 9 blocks, with different colour patterns on each side. With the blocks the patients had to build a predetermined pattern within a restricted period of time. The sum score ranges from 0 to 51 points; lower values are indicating more severe visuospatial disabilities.

*Verbal Digit Span* was applied to measure short-term memory. This test is a subtest of the Wechsler Memory Scale German adaption [27]. The examiner reads a series of digits aloud which have to be repeated by the subject afterwards. Each correctly repeated series granted a point, adding up to a sum score ranging from 0 to 12 points, where higher values indicate better short-term memory performance.

### 2.3. EEG Data

During 15 min an eyes-closed, resting-state, 256-channel EEG was recorded (Netstation 300; EGI Inc., Eugene, Oregon, USA). The reference electrode was Cz and rereferenced to the average. The sampling frequency was 1 kHz. Segments of >35 s without artifacts or signs of sleep were visually selected. EEGs were filtered (2,500 order least-square filter; band pass: 0.5–70 Hz, notch: 50 Hz) and bad electrodes were automatically detected (using TAPEEG software) [19] and visually checked for plausibility. Artifacts such as ECG and eye blinks were detected and removed by an application of an independent component analysis. Channels with bad activation were interpolated (spherical spline method). Frequency analysis was performed with the “Welch”-method [37]. Sliding windows of 4 s with 80% Hanning windows and the detection of bad windows were analysed with automated routines [19]. Semiautomatic processing of the data was applied in order to calculate the relative power in alpha (8–18 Hz) and theta (4–8 Hz) frequency bands across the 10 brain regions (see Figure 4). Relative alpha/theta ratios were calculated from the frequency results.

### 2.4. Statistical Procedure

The R software version 3.2.3 was used for statistical analysis [38]. The level of statistical significance was set at *p* = .05.

A linear mixed effects model (LME) with the alpha/theta ratio as the dependent variable was used to test the association between EEG slowing and visuospatial test scores. The test performance was used as fixed factor and the patients as random factor. Consequently, a *b*-value below zero indicates that the worse the alpha/theta ratio, the lower the test performance. The LME is a linear model that allows repeated measurement. This model was adopted due to the repeated measurements, resulting from EEG electrode subdivision into the five brain areas. An exhaustive search, according to Stöcklin [39], with age, gender, years of education, motor symptoms (UPDRS III), disease duration, depression scale (BDI), MMSE, and LED showed that gender and age were confounding factors for alpha/theta ratio. The assumptions for LME are homoscedasticity (homogeneous variance), linearity, no influential data points, and independence (collinearity). The plot of the standardized residuals showed a heterogeneous variance relating to the fitted values. A logarithmic transformation was performed in order to achieve a normal distribution as proposed by Crawley [40]. After the logarithmic transformation the residuals in the used LME models were normally distributed around zero and therefore the requested homogeneous variance was achieved [41]. Plots of the random effects showed an unsystematic arrangement around zero. This confirmed a normal distribution of the errors (linearity) [41]. Influential data points were not found. Furthermore, there was no correlation between the predictor variables.

In a first step, the LME calculations showed no correlation between alpha/theta ratio and the task performance. Because of this finding, a median split was used to separate potentially clinically conspicuous from inconspicuous patients in regard to the visuospatial ability. The median split was calculated separately for each neuropsychological test. Group A included patients from the lowest tasks performance up to the median and group B included patients from the median up to the best tasks performance. Clinical and demographic variables between the median split groups were analysed by means of *X*^2^-test or Mann–Whitney *U*-test as appropriate. The difference in relative alpha/theta ratio between the left and the right cerebral hemisphere was calculated by a Wilcoxon's matched-pairs signed rank test. There were no significant differences in the relative alpha/theta ratio between the right- and left-sided electrode in the PD-patients (*p* = .316). Therefore, the analyses were based on combined data of the alpha/theta ratio for the right- and left-sided electrode locations. Furthermore, to compare the results, the LME were calculated with *z*-scaled Block Design and the Digit Span scores.

## 3. Results

The visuospatial decrease which would be expected in PD-patients was weak in this population (see Table 1). The descriptive statistics of the clinical performance, split in the two median groups A and B, are shown in Table 2. Significant differences between group A and B had been obtained in the Clock Drawing Test with regard to the MMSE and the BDI and in the Digit Span with regard to the disease duration. Otherwise there were no significant differences between the groups. The exhaustive search had shown that gender and age were confounding factors for all used neuropsychological tests. The EEG alpha/theta ratio was different between males and females in all areas [parietal *U*(57/57) = 211, *p* = .024, frontal *U*(57/57) = 200, *p* = .014, central *U*(57/57) = 205, *p* = .018, temporal, *U*(57/57) = 210, *p* = .023, and occipital *U*(57/57) = 194, *p* = .010].

### 3.1. Clock Drawing

The LME results for the Clock Drawing Test are shown in Table 3. A significant lower alpha/theta ratio was recognised in PD-patients with an incorrectly drawn clock compared to PD-patients, who had produced a correctly drawn clock. The group difference was more distinct in parietal areas than in central, temporal, and occipital areas.

### 3.2. ROCF

As shown in Table 4, in group A of the ROCF, the results revealed that the deeper the parietal alpha/theta ratio the worse the ROCF performance. An increase of 1.0 *z*-score in the ROCF increased the parietal alpha/theta ratio by *b* = 0.59, *t*(24) = 2.73, and *p* = .012. There was also a significant positive association between occipital alpha/theta ratio and the ROCF performance in the ROCF group A. An increase of 1.0 *z*-score in the ROCF increased the occipital alpha/theta ratio [*b* = 0.50, *t*(24) = 2.31, *p* = .030]. No significant association was found in the other cortical areas (see Table 4). Furthermore, the associations in the parietal areas were different from the frontal [*b* = −.19, *t*(104) = −2.28, and *p* = .025], central [*b* = −.24, *t*(104) = −2.88, and *p* = .005], and temporal [*b* = −.24, *t*(104) = −2.88, and *p* = .005] areas. There was no significant difference between the association in parietal areas and the association in occipital areas [*b* = −.09, *t*(104) = −1.06, and *p* = .290]. In the ROCF group B there was neither an association between alpha/theta ratio and the ROCF *z*-score nor a significant difference between the association in parietal areas and the associations in the remaining areas.

### 3.3. Block Design Test

The LME results for the Block Design Test are shown in Table 5. No significant correlation was found between the alpha/theta ratio and the Block Design performance. In the Block Design group A there was simply a tendency towards a positive correlation between the parietal alpha/theta ratio and the Block Design performance. An increase of 1.0 *z*-score in the Block Design Test increased the alpha/theta ratio [*b* = 0.48, *t*(25) = 1.96, and *p* = .062]. No associations were found in the other cortical areas. The association in the parietal areas differed from the association in temporal areas [*b* = −.24, *t*(108) = −2.54, and *p* = .013]. Furthermore, there was a trend towards a difference between the association in parietal areas and the association in frontal [*b* = −.16, *t*(108) = −1.71, and *p* = .090] and central areas [*b* = −.16, *t*(108) = −1.68, and *p* = .096].

### 3.4. Verbal Digit Span forward

The results in both Digit Span groups, A and B, showed no correlation between the alpha/theta ratio and the Digit Span performance (see Table 6). However, in the Digit Span group A a slight tendency towards a negative correlation between the alpha/theta ratio and the Digit Span performance was observable in frontal [*b* = −.35, *t*(25) = −2.05, and *p* = .051], central, [*b* = −.29, *t*(25) = −1.73, and *p* = .096], and parietal areas, [*b* = −.34, *t*(25) = −2.02, and *p* = .054]. There were no associations in the other cortical areas. Furthermore, the association in the parietal areas was different from the occipital areas, [*b* = .14, *t*(108) = 2.21, and *p* = .030] in group A. In group B a difference between the association in parietal areas and the association in central areas, [*b* = −.16, *t*(104) = −2.07, and *p* = .041] was observable (see Table 6).

## 4. Discussion

The aim of this study was to investigate possible relationships between parietal and occipital EEG slowing and visuospatial deficit in nondemented PD-patients. The EEG slowing was measured by determining the alpha/theta ratio in the frontal, central, temporal, parietal, and occipital lobe. The visuospatial ability was assessed by three different neuropsychological tests: Clock Drawing Test, ROCF, and Block Design Test. A LME was used to explore the association between visuospatial performances and alpha/theta ratio.

In contrast to previous findings, the PD-patients in our study showed only slight deficits in visuospatial ability [3, 4]. This might be explained by the high education level of the patients in our sample. Recent studies indicated that a high education is predictive for a slower cognitive decline [42–44]. In order to separate potentially clinically conspicuous from inconspicuous patients in regard to the visuospatial ability a median split was used.

The results of this study show that PD-patients with a parietal EEG slowing manifest a visuospatial deficit. This result is in line with findings from voxel-based morphometry MRI analysis, indicating correlations between visuospatial ability in PDD-patients and changes in the occipitotemporal and dorsoparietal cortices in comparison to healthy controls [11]. Nombela et al. [45] also reported a correlation between parietal activity and visuospatial performance. In line with our hypothesis, the association between the EEG slowing and the visuospatial task performance is particularly pronounced in parietal areas compared to frontal, central, and temporal areas (see Figure 3). In addition, no differences between the association in parietal and occipital areas were detected in our sample. This finding indicates that the association is not explained by the global EEG slowing, as has been shown in previous studies in patients with PD [46, 47]. Though other previous studies also indicated that the visuospatial ability is not correlated with global EEG slowing measured by median frequency [48], more research is needed to substantiate this point. Our present findings are also in line with the dual-stream hypothesis of the visual processing claiming the occipitoparietal projection system to be responsible for visuospatial performance [12].

In all groups with test scores above the median (i.e., unimpaired visuospatial abilities), no correlations were found between the alpha/theta ratio and the task performances, indicating that a relationship between the visuospatial ability and the EEG is only measurable if the visuospatial ability score decreases below the median.

In contrast to the results of the ROCF and the Block Design Test, the results of the Clock Drawing Test showed that PD-patients drawing an incorrect clock had lower alpha/theta ratio not only in parietal and occipital brain areas but also in all other brain areas. PD-patients with a flawless CDT-performance did not show this association (see Figure 2). The neuroanatomical correlates of Clock Drawing Test performance were investigated in several studies, but the findings are inconsistent [49–52]. This discrepancy might probably stem from the fact that the Clock Drawing Test measures also executive function, numerical and verbal memory, and visuospatial ability [34, 53]. Furthermore, Matsuoka et al. [52] explored the relationship between regional cerebral blood flow and different scoring criteria of the Clock Drawing Test in patients with Alzheimer's disease, revealing that different criteria correlate with different brain regions. Consequently, it can be concluded that for a correlative analysis between different brain areas and CDT-performance an overall classification into errorless and incorrect CDT-performance might be too simple. Hence, future studies should adopt differential scoring CDT-scoring criteria to unravel this relationship.

In our study, the results of the ROCF and the Block Design Test are consistent. In line with our findings, for both tests, neuroanatomical correlations in parietal and occipital areas were also found in previous studies [54–57]. However, the results are more specific in ROCF than in the Block Design Test. This result could be partly explained by the somewhat different cognitive processes required by the different tasks. Hence, while the ROCF is mainly a visuoconstructive task, with a preponderance on visuoperceptive, visuospatial as well as graphomotor abilities without time limitation, the Block Design Test on the other hand requires mental rotation as well as geometric fragmentation analysis under time-restriction [26].

The Digit Span measures working memory. Studies on healthy subjects, using either transcranial magnetic stimulation [58] or functional neuroimaging [59], were able to show an involvement of the right dorsolateral prefrontal cortex in Digit Span processing. Furthermore, Gerton et al. [59] reported that parietal and occipital areas are activated during the Digit Span forward task. In the present study no association was found between EEG slowing and the Digit Span performance. Nevertheless, a slight tendency towards a negative correlation between the alpha/theta ratio and the Digit Span performance was observed in frontal, central, and parietal areas (see Figure 3). The involvement of parietal areas could be explained by the use of the visual imagination strategies the subjects used during the Digit Span test [34]. An explanation for the negative tendency could be that the Digit Span performance is not relating to EEG slowing caused by a shifting in alpha/theta ratio but by a shifting in others frequency range (e.g., theta/delta ratio or beta/alpha).

The results from our present study do not reveal significant differences regarding confounding factors between the median split groups of the ROCF and Block Design Test. However, there were significant differences between the subgroups according to their Clock Drawing Test performance. Patients with an incorrectly drawn clock had a lower MMSE score than patients with a flawless CDT-performance. This finding is not surprising since both CDT and MMSE are also measures of global cognitive dysfunction [28–30, 60]. In addition, many studies found a correlation between these two tests [53, 61–63]. Furthermore, PD- Patients with an incorrectly drawn clock had a lower BDI score than PD-patients with a correctly drawn clock. These findings were unexpected as it is well-known that there is an association between depression and cognitive performance [64]. The results exploring the association between severity of depression and the performance in the Clock Drawing Test are inconsistent. Some authors have reported a significant negative relation [65, 66], whereas others have found minimal or no effect between the severity of depression and the Clock Drawing performance [61, 67–70]. In the present study, the BDI score has no significant influence on the used LME model. Nevertheless, the results cannot predict whether severity of depression has an influence on the cognitive performance. Another group difference is found between Digit Span performance and disease duration. Patients in the Digit Span group A have a shorter disease duration than patients in the Digit Span group B. This result contrasts with some recent findings which have shown a reduction of working memory capacity in PD-patients as the disease progresses [71, 72]. Hence, our result might be caused by the sampling process based on a right-skewed sample..

**Figure 1:**
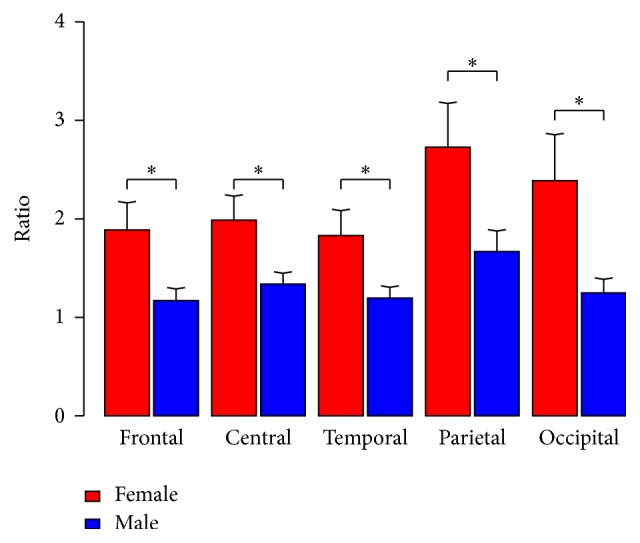
Difference alpha/theta ratio between gender in the different brain areas. ^*∗*^*p* < .050.

**Figure 2:**
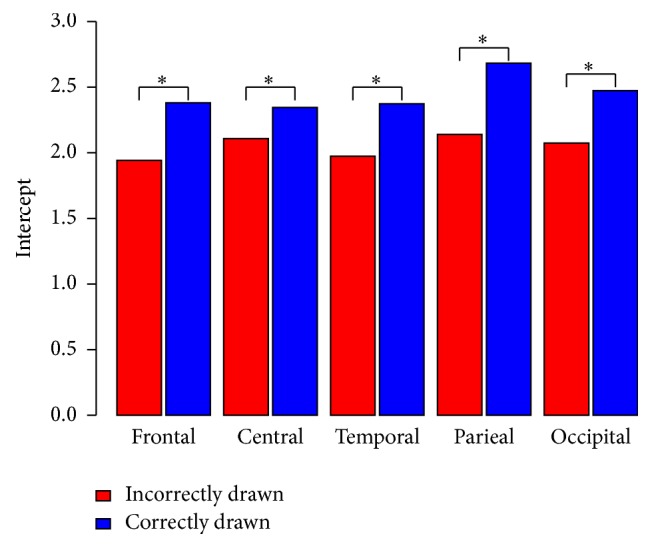
Comparison intercept between incorrectly and correctly drawn Clock Drawing Test groups related to the alpha/theta ratio in the different brain areas. ^*∗*^*p* < .050.

**Figure 3:**
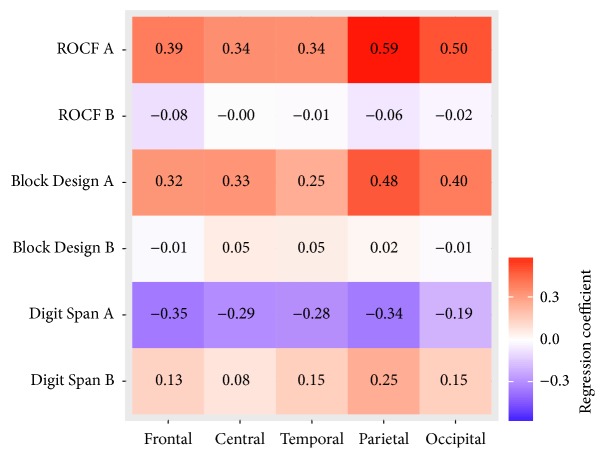
Correlation between alpha/theta ratio and tasks performance in different brain areas; ROCF is Rey-Osterrieth Complex Figure Test; A is group with lower tasks performance; B is group with higher tasks performance.

**Figure 4:**
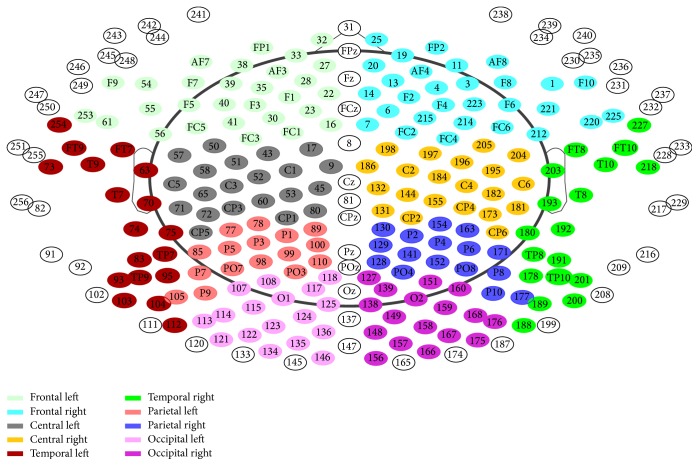
Electrodes allocation for frequency analyse [19].

While in the present study gender (see Figure 1) and age are identified as confounding factors and were consequently controlled in the LME, other authors have found only a small influence for these variables on the EEG activity [46, 73]. Hence, further studies are needed to determine the influence of gender and age on EEG slowing in PD-patients.

One limitation of our study is that the calculation of *z*-scores for the ROCF was based on a norm population whereas the calculations of *z*-scores for the Block Design Test and the Digit Span were based on our study population, limiting a comparison of the tests. Moreover, since only 17 of 57 patients in our sample were female, the gender influence on EEG limits a generalization. Although the unequal distribution of gender is well-known in PD [74, 75] an equal gender distribution should be considered in future studies. Another limitation of this study is that there were no healthy controls included. Therefore, the conclusion that the findings are specific for patients with PD cannot be drawn. Moreover, since we adopted a priori hypothesis based models, we relinquished to account for multiple comparisons. Therefore the interpretation of the results should be treated with caution, bearing in mind that the probability of correlative findings increases with the number of tests performed. In conclusion, in PD-patients with only slight deficits in visuospatial abilities the visuospatial performance is related to parietal and occipital EEG slowing. The association between the EEG slowing and the visuospatial task performance is particularly pronounced in parietal areas compared to frontal, central, and temporal areas.

## Figures and Tables

**Table 1:** Descriptive statistics and tasks performance of total group.

Parkinson patient *N* = 57	M	SD
Sex (M/F)	40/17	
Age (years)	67.21	(6.96)
Education (years)	14.67	(3.01)
UPDRS III	14.77	(11.13)
MMSE	28.70	(1.06)
Disease duration (years)	5.25	(0.50)
Dose of L-dopa (mg/day)	597.60	(372.06)
BDI	7.22	(4.47)
Clock Drawing Test (incorrectly/correctly drawn)	16/41	
ROCF	28.83	(4.19)
Block Design Test	24.79	(7.56)
Verbal Digit Span forward	7.49	(1.72)

*Note*. Means and standard deviations relate to raw values. UPDRS III = Unified Parkinson's Disease Rating Scale subscale III (range 0–108); MMES = Mini-Mental State Examination (range 0–30); BDI = Beck Depression Inventory (range 0–63); ROCF = Rey-Osterrieth Complex Figure Test.

**Table 2:** Descriptive statistics of median split groups.

	Clock Drawing Test			ROCF			Block Design Test			Digit Span		
	Incorrectly drawn	Correctly drawn		A	B		A	B		A	B	
	*N* = 16	*N* = 41		*N* = 26	*N* = 29		*N* = 29	*N* = 27		*N* = 29	*N* = 28	
Allocation	Median	Median	*p*	Median	Median	*p*	Median	Median	*p*	Median	Median	*p*
Sex (M/F)	11/5	29/12	1.000	19/9	20/7	.833	20/9	19/8	1.000	20/9	20/8	1.000
Age	67.5	67	.930	66.5	69.0	.295	67.0	69.0	.384	67.0	67.5	.994
Education	15	15	.964	14	15	.572	14	15	.092^.^	15	15	.413
UPDRS III	13.5	13.5	.765	17.0	10.0	.166	15.5	13.0	.511	14.0	13.5	.818
MMSE	28.5	29	.036^*∗*^	29	29	.347	29	29	.209	29	29	.322
Disease duration	5.27	3.37	.160	4.30	3.24	.508	4.12	3.37	.558	2.94	4.74	.038^*∗*^
Dose of L-dopa	666	510	.247	650	495	.206	590	550	.906	510	585	.296
BDI	4.5	8.0	.024^*∗*^	6.5	7.18	.901	6.5	7.35	.655	7	7	.941

*Note*. Values are expressed by median; UPDRS III = Unified Parkinson's Disease Rating Scale subscale III (range 0–108); MMES = Mini-Mental State Examination (range 0–30); BDI = Beck Depression Inventory (range 0–63); ROCF = Rey-Osterrieth Complex Figure Test; A = group with lower tasks performance; B = group with higher tasks performance; ^*∗*^*p* < .050, ^.^*p* < .1.

**Table 3:** Correlation between alpha/theta ratio and Clock Drawing Test.

	Δ incorrectly and correctly drawn		Comparison *b* parietal/other areas
Brain area	*b*	*p*	*b*
Parietal	0.54 (0.18)	.003^*∗*^	
Frontal	0.44 (0.18)	.016^*∗*^	0.134
Central	0.40 (0.18)	.025^*∗*^	0.046^*∗*^
Temporal	0.40 (0.18)	.027^*∗*^	0.040^*∗*^
Occipital	0.36 (0.18)	.045^*∗*^	0.009^*∗*^

*Note*. *b* = beta coefficient (standard errors); using a linear mixed effects model (LME); ^*∗*^*p* < .05.

**Table 4:** Correlation between alpha/theta ratio and Rey-Osterrieth Complex Figure Test.

	ROCF A			ROCF B		
brain areas	*b*	*p*	Comparison *b* parietal/other areas	*b*	*p*	Comparison *b* parietal/other areas
			*p*			*p*
Parietal	0.59 (0.21)	.012^*∗*^		−0.06 (0.17)	.738	
Frontal	0.39 (0.21)	.079^.^	.025^*∗*^	−0.08 (0.17)	.653	.724
Central	0.34 (0.21)	.123	.005^*∗*^	−0.00 (0.17)	.984	.337
Temporal	0.34 (0.21)	.123	.005^*∗*^	−0.01 (0.17)	.967	.372
Occipital	0.50 (0.21)	.030^*∗*^	.290	−0.02 (0.17)	.900	.530

*Note*. *b* = beta coefficient (standard errors); using a linear mixed effects model (LME); ^*∗*^*p* < .05, ^.^*p* < .1.

**Table 5:** Correlation between alpha/theta ratio and Block Design Test.

	Block Design A			Block Design B		
brain areas	*b*	*p*	Comparison *b *parietal/other areas	*b*	*p*	Comparison *b *parietal/other areas
			*p*			*p*
Parietal	0.49 (0.25)	.062^.^		0.02 (0.15)	.886	
Frontal	0.32 (0.25)	.202	.090^.^	−0.01 (0.15)	.938	.588
Central	0.33 (0.25)	.198	.096^.^	0.05 (0.15)	.711	.578
Temporal	0.25 (0.25)	.328	.013^*∗*^	0.05 (0.15)	.735	.631
Occipital	0.40 (0.25)	.121	.353	−0.01 (0.15)	.962	.640

*Note*. *b* = beta coefficient (standard errors); using a linear mixed effects model (LME); ^*∗*^*p* < .05, ^.^*p* < .1.

**Table 6:** Correlation between alpha/theta ratio and verbal Digit Span forward.

	Digit Span A			Digit Span B		
Brain areas	*b*	*p*	Comparison *b* parietal/other areas	*b*	*p*	Comparison *b* parietal/other areas
			*p*			*p*
Parietal	−0.34 (0.17)	.054^.^		0.25 (0.22)	.272	
Frontal	−0.35 (0.17)	.051^.^	.944	0.13 (0.22)	.548	.156
Central	−0.29 (0.17)	.096^.^	.469	0.08 (0.22)	.707	.041^*∗*^
Temporal	−0.28 (0.17)	.107	.381	0.15 (0.22)	.505	.218
Occipital	−0.19 (0.17)	.265	.030^*∗*^	0.13 (0.22)	.510	.210

*Note*. *b* = beta coefficient (standard errors); using a linear mixed effects model (LME); ^*∗*^*p* < .05, ^.^*p* < .1.
